# Breakthrough cancer pain management (BTcP)—gap analysis of the current Australian landscape

**DOI:** 10.1007/s00520-026-10518-z

**Published:** 2026-03-11

**Authors:** Wei Lee, Ahmed Nagla, Aaron Bak Ong Wong, Linda Magann, Melanie Lovell, Edward Mantle, Kate Reed, Ghauri Aggarwal, Aaron K. Wong, Chris Pene, Peter Allcroft, Gregory B. Crawford, Natasha Michael

**Affiliations:** 1 University of Sydney, Camperdown, NSW Australia; 2 MQHealth Clinical Trial Unit, Macquarie University, New South Wales, Australia; 3 Peter MacCallum Cancer Centre, Melbourne, VIC Australia; 4 Sir Peter MacCallum Department of Oncology, The University of Melbourne, Victoria, Australia; 5 Eastern Health, Wantirna, VIC Australia; 6 Monash University, Melbourne, VIC Australia; 7 St George Hospital, Kogarah, NSW Australia; 8 HammondCare, Greenwich Hospital, Greenwich, NSW Australia; 9 Gold Coast University Hospital, Southport, QLD Australia; 10 Robina Hospital, Robina, QLD Australia; 11 Canberra Hospital, Australian Capital Territory, Garran, Australia; 12 Concord Repatriation General Hospital, Concord, NSW Australia; 13 The Royal Melbourne Hospital, Melbourne, VIC Australia; 14 St Vincent’s Hospital, Darlinghurst, NSW Australia; 15 Southern Adelaide Palliative Services, Bedford Park, South Australia Australia; 16 School of Medicine, College of Health, Adelaide University, Adelaide, Australia; 17 Institute of Health Research, University of Notre Dame Australia, Fremantle, WA Australia; 18 Plunkett Centre for Ethics, Bendigo Health, Victoria, Australia

**Keywords:** Breakthrough cancer pain, Cancer pain, Opioids, Pain management, Palliative care, Research gaps

## Abstract

**Purpose:**

Breakthrough cancer pain (BTcP) is an evolving clinical challenge, with limited guideline-specific direction. This study aimed to identify gaps in breakthrough cancer pain (BTcP) diagnosis and management in Australia and propose practical, evidence-informed actions to improve assessment, prescribing and equitable access to effective analgesia.

**Methods:**

A gap analysis was conducted between September 2023 and September 2024, using three hybrid roundtable meetings involving 13 medical and nursing clinicians and researchers. Participants were selected for expertise in BTcP, including rapid-onset opioids (ROOs) policy development, BTcP research and education. A targeted review of the literature and guidelines framed the discussions. Meetings were recorded, transcribed and iteratively member-checked; thematic synthesis identified key gaps and potential solutions.

**Results:**

Five interrelated gaps were identified: (1) inconsistent definitions of BTcP undermining case identification and research comparability; (2) assessment and measurement gaps with uptake of validated tools limited by perceived respondent burden and clinical utility; (3) heterogeneous approach to BTcP with limited comparative evidence guiding ROOs versus immediate-release opioid use and dosing strategies; (4) implementation and systems barriers including workflow, prescribing complexity and clinician training needs; (5) equity in opioid supply and restricted access to vulnerable populations. Recommended actions include Delphi consensus on definition, development and validation of subtype-sensitive assessment tools, pragmatic comparative effectiveness and implementation studies, co-designed prescribing templates and stakeholder engagement to address supply chain and regulatory barriers.

**Conclusions:**

Sequential, coordinated efforts—consensus building, measurement development, targeted research, co-designed implementation supports and supply chain planning—are required to advance equitable, evidence-based BTcP care in Australia.

## Background

Cancer pain remains one of the most common and debilitating symptoms experienced by individuals with cancer, affecting approximately one in two patients [1], yet it continues to be undertreated in nearly one in three [2]. Up to 70% of patients with advanced cancer suffer moderate to severe pain [3], with younger individuals more likely than older individuals to report cancer-related pain and episodic flares [4]. The International Classification of Diseases 11th revision (ICD-11) now classifies cancer pain under chronic cancer–related pain, encompassing pain arising from the primary tumour, metastasis or cancer treatments such as radiotherapy or surgery [5]. Importantly, ICD-11 distinguishes the temporal characteristics of cancer pain into continuous background pain and intermittent episodic pain, the latter commonly referred to as breakthrough cancer pain (BTcP). Although the definition of BTcP has evolved over the past two decades, it is widely described as a transitory exacerbation of pain that occurs despite otherwise stable and adequately controlled background pain, arising either spontaneously (unpredictably) or incidentally (predictably) [6].

BTcP is both common and debilitating, affecting an estimated 40–80% of cancer patients [7]. When experienced, BTcP disrupts multiple dimensions of life beyond pain itself, including daily functioning, work and sleep [8]. It is characterised by its rapid-onset (typically reaching a peak within 10 min and short duration usually lasting 30–60 min), varying underlying pain mechanism, and may follow a circadian rhythm, being most prevalent in late morning [9, 10]. Presentation differs across cancer types and clinical settings, appearing more predictable in head and neck cancers than other tumours [11]. Food ingestion is a recognised trigger for BTcP among patients with head and neck cancer, likely due to mucositis, and among those with visceral abdominal malignancies such as pancreatic cancer [11, 12]. An Italian study of 1206 females with breast and gynaecological cancer found a later onset of BTcP when compared to non–female-specific cancers and a higher prevalence of non-predictable BTcP in gynaecological cancers [13].

This heterogeneity in the temporal pattern and mechanisms of BTcP makes it challenging to apply a standardised analgesic approach using conventional immediate-release oral opioids [14, 15]. As the primary goal is to provide rapid relief that aligns the pharmacokinetics of the medication with the temporal characteristics of BTcP, rapid-onset opioids (ROOs), delivered as transmucosal, sublingual or intranasal formulations, have emerged as the preferred treatment option [16]. Over the past decade, there has been an increasing research focus on the efficacy of ROOs, particularly fentanyl-based formulations, in managing BTcP [17], with a systematic review demonstrating evidence particularly for the use of transmucosal ROO and alternative oral opioids dosed proportionally to baseline opioids [18]. Notably, a longitudinal study involving 230 cancer patients found that establishing personalised BTcP goals facilitated the achievement of meaningful pain relief by enabling more individualised care and tailored assessment of treatment response [19].

Despite its high prevalence and associated morbidity, a review of BTcP and international cancer pain guidelines indicates a continued reliance on expert opinion rather than robust research when recommending approaches to the identification, assessment and management of BTcP [20, 21]. The persistent endorsement of oral opioids may reflect ongoing uncertainty regarding the role and availability of ROOs [22–25]. However, recent consensus recommendations from 107 cancer experts in Spain recognised procedural BTcP as a distinct subtype arising during diagnostic or therapeutic interventions and highlighted the importance of premedication with an appropriate ROOs [26].

Despite recognition of its clinical significance, research examining clinicians’ and patients’ perspectives on BTcP remains limited. Studies highlight persistent challenges related to communication, definition, impact and effective pain relief [27]. A Turkish study involving 148 oncology nurses reported limited awareness and understanding of BTcP, particularly regarding its distinction from baseline pain [28]. Similarly, a recent Australian study emphasised the need for adequate resourcing, targeted training and interdisciplinary collaborations to optimise care in BTcP [29].

To advance the appreciation of BTcP in the Australian context, it is important to systematically characterise current gaps and challenges in clinical practice. The care of individuals with BTcP needs to be formally characterised. This study aimed to identify key barriers to the diagnosis and management of BTcP and to propose practical, actionable steps toward potential solutions, beginning in Australia, with the broader aim of contributing to international efforts to enhance cancer pain management globally.

## Methods

A gap analysis was undertaken using the principles of Levin and Lauder (2013), exploring the current state of care delivery practices and the target state of best evidence-based practices, identifying the gap between the two and generating potential solutions [30].

### Formation of and conduct of the working group

A working group was convened by the chair and comprised senior palliative care clinicians and researchers from medical and nursing backgrounds. Participants were selected based on one or more of the following criteria: (a) involvement in a working party for listing ROOs on the Australian Pharmaceutical Benefits Scheme; (b) academic publications on BTcP or related aspects of cancer pain (national or international); (c) participation in local or national education programs on BTcP or ROOs use; (d) contribution to national or international policy or guideline development for cancer pain management; (e) recommendation by professional bodies (e.g. Cancer Symptom Trials, Palliative Care Australia). Representations were sought from diverse healthcare services and settings across Australia, and formal invitations were extended by the chair. Participants were reimbursed for their travel and time spent attending meetings.

Three roundtable meetings were held over a 12-month period (September 2023 to September 2024). Meetings were conducted both face-to-face and online, and each session lasted approximately 3 hours. At each meeting, a summary of the current evidence on BTcP, derived from formal literature and a review of national and international policies and guidelines, was presented to frame discussion of the prevailing clinical landscape. Facilitated by the chair, the group then considered the ideal target state for BTcP care in the Australian context, identified perceived gaps and barriers to optimal assessment and management and developed recommendations for next steps, with an eye to future international application.

Meetings were recorded and transcribed by an independent administrative support person. Detailed notes and minutes were circulated to working group members after each meeting for verification and amendment. The notes from all three meetings were analysed and synthesised by WL, and the resulting synthesis was circulated to all members for further comments and verification. Consensus was achieved when all members of the working group endorsed a recommendation.

## Results

The demographics of the 13 participating working group members who accepted invitations and attended meetings are illustrated in Table 1. The majority were male, medical, working in metropolitan public settings, had formal affiliations with an Australian University, and all participants had worked in palliative care for 10 or more years.

**Table 1:** Socio-demographics of participants (*n* = 13)

Items	*N* (%)
**Age in years** (mean, range)	52.4 (39–69)
**Gender**	
Male	8 (61.5)
Female	5 (38.5)
**Profession**	
Medical	11 (84.6)
Nursing	3 (23.1)
**Location of work**	
Metropolitan	12 (92.3)
Regional/rural	2 (15.4)
Service setting^*^	
Acute hospital	12 (92.3)
Subacute hospital	7 (53.8)
Community/outpatient	9 (69.2)
Service type^*^	
University	11 (84.6)
Public hospital/healthcare entity	13 (100)
Private hospital/healthcare entity	5 (38.5)
Community or primary care	4 (30.8)

^*^Some participants worked across more than a single service setting and type

The gap analysis revealed heterogeneity in the perceived optimal approach to BTcP, with five interrelated gaps, as detailed below and in Table 2.

**Table 2:** Gap analysis of the current landscape of breakthrough cancer pain management in Australia

Current state	Proposed actions	Target state
Inconsistent definitions of BTcP	Expert consultations using the Delphi method to facilitate a consensus definition of BTcP	A consensus definition of BTcP to facilitate standardise treatment and research
Paucity of validated BTcP-specific screening and assessment methods	Conducting qualitative studies to develop screening and assessment methods that explore BTcP features and differentiate analgesic response. These methods should be tested along with context-appropriate validation in clinical studies	Standardised screening and assessment methods that incorporate specific BTcP subtypes to allow standardised recommendations of analgesic options
Heterogeneity in opioid utilisation in BTcP	Develop evidence to guide BTcP management	A consensus BTcP management guideline
(a) Limited data on clinician perspectives and approaches to BTcP	(a) Explore clinician perspectives and approaches to management of BTcP and postulate strategies to improve care via relevant professional bodies	(a) Establish clinician approaches towards BTcP, including key barriers to care
(b) Limited understanding of the respective roles of conventional immediate-release opioids and rapid-onset opioids in BTcP management	(b) Study the analgesic response and tolerability of different analgesics for BTcP; compare proportional versus titration dosing regimens for rapid-onset opioids; explore predictors of positive response, toxicity and equianalgesic dosing	(b) Consensus guidelines addressing the role of various opioid types and non-opioid adjuncts for different BTcP subtypes
(c) Limited evidence on BTcP in vulnerable populations (older adults, culturally and linguistically diverse populations, individuals experiencing homelessness and substance misuse disorders)	(c) Explore strategies to support vulnerable populations in BTcP research through consumer and caregiver engagement and their inclusion in research	(c) Adapted approaches inclusive of vulnerable populations in guidelines for BTcP management
(d) No method to screen, assess and care for individuals at risk of opioid misuse when experiencing BTcP	(d) Interdisciplinary qualitative research involving consumers and clinicians across palliative medicine, chronic pain and addiction medicine specialties to define the construct of opioid misuse in advanced cancer	(d) Validated screening and assessment methods to identify people at risk of opioid misuse when experiencing BTcP
Barriers to implementation of rapid-onset opioid titration methods	Develop a quality improvement process that facilitates a specialised prescribing form (paper and electronic) for the titration of rapid-onset opioids through co-design with clinicians, informatics, industry and regulatory bodies	Written and electronic prescribing resources that support the titration of rapid-onset opioids, conforming to relevant regulatory requirements
Variability of access to required opioids due to supply issues, and fragmented communication between industry, professional bodies and clinicians	Explore strategies for better engagement involving representatives from palliative care professional bodies, research collaborative groups, relevant pharmaceutical industry, government regulatory bodies, health services and pharmacies to achieve the target state, devising contingency plans for essential medicine shortages	Consistent access to opioids for BTcP; coordinated liaison strategies between relevant stakeholders around opioid supply chain issues to ensure early contingency planning

Abbreviation: *BTcP* - breakthrough cancer pain

### Inconsistent definition of BTcP

The working group reviewed existing definitions of BTcP and noted that, internationally, the construct is heterogeneous and has evolved over time. It recognised that many clinical guidance documents in Australia do not currently define BTcP. Members acknowledge the persistent difficulty in distinguishing breakthrough pain from end-of-dose failure or uncontrolled persistent background pain in the clinical context and how such definitional variability likely contributes to the wide variance in reported prevalence of BTcP.

The group concluded that establishing a consistent, context-appropriate definition of BTcP in Australia and incorporating it into guidelines and policies is essential to improve identification, assessment and management. Achieving this will require structured expert consultation with relevant professional bodies (e.g. the Australian and New Zealand Society of Palliative Medicine, the Australian Pain Society and the Clinical Oncology Society of Australia) and could be informed by qualitative studies, followed by consensus-building methods such as the Delphi technique. Further implementation studies exploring strategies to ensure consistent use of BTcP definitions across the literature were recommended.

### Assessment and measurement gaps

The group noted that several validated instruments exist for screening and assessing cancer-related pain in clinical and research settings, including the Symptom Assessment Scale (SAS) [31], the Palliative Care Outcome Scale (POS) [32] and the Brief Pain Inventory (BPI) [33]. However, these measures were not developed specifically to detect or characterise BTcP. The group recognised that to date, the Breakthrough Pain Assessment Tool (BAT) remains the only validated instrument designed specifically for BTcP [34]. Despite its specificity, the adoption of the BAT into routine clinical use across Australia has been impeded by concerns about respondent burden and perceptions that the tool does not directly inform analgesic selection.

The group concluded that future development of BTcP assessment tools should enable clear differentiation between actual BTcP and its subtypes, from end-of-dose failure or poorly controlled background pain, as this differentiation can inform more precise, mechanism-based analgesic recommendations tailored to the pain characteristics. This would facilitate rapid-onset, short-duration incident pain to be more appropriately managed with ROOs than with immediate-release opioids. This differentiation would also allow for the appropriate titration of inappropriate background analgesia when required. The group further discussed incorporating emerging pharmacogenomic assessments to further refine opioid selection by identifying likely responders and those at increased risk of adverse effects. These approaches should be evaluated in clinical trials that also examine inter-individual variability in opioid metabolism and its impact on efficacy and safety.

### Differences in approaches to BTcP

The general consensus among group members was that clinician perspectives and practices regarding BTcP in Australia are poorly characterised and appear to vary regionally. More substantive targeted surveys and qualitative work with professional bodies are needed to map current approaches. Furthermore, the group judged that comparative data on rapid-onset opioids (ROOs) versus conventional opioids were insufficient to guide routine practice. Clinician hesitancy may be related to the lack of accepted ROO–morphine conversion ratios and uncertainty remains about whether proportional or titration dosing is preferable. As guidelines differ, some group members cautioned against first-line ROOs use while others favoured ROOs for incident, short-duration pain. Harmonised, regulator-aligned guidance would help clinicians balance efficacy, cost and practicality of prescribing.

### Implementation and system barriers

The group recognised that implementing interventions for BTcP can be challenging. Although ROOs, such as sublingual fentanyl citrate, are an important therapeutic option, many services report logistical barriers to initiating ROOs because of complex titration requirements. This complexity requires structured quality improvement processes and a multidisciplinary approach that involves physicians, nurses, pharmacists and regulatory bodies. Standardised adaptable forms, instructions and charting, compatible with both paper and electronic prescribing systems, should be developed, together with clinician training in their use. Expert consensus on nomenclature and indications that align with regulatory requirements is essential. Written guidance should combine clear instructional and directive elements and include reference algorithms to support prescribers and medication administrators.

### Equity and vulnerable populations

The group identified significant barriers to opioid access that impede optimal BTcP management. Recently, Australia-wide shortages of commonly used agents for managing BTcP, including immediate-release oral morphine, oxycodone and hydromorphone, were attributed in part to industry and supply chain disruptions. Furthermore, access to ROOs was hampered in many acute hospitals and in regional and remote settings due to cost and limited availability. Finally, it was recognised that evidence to guide BTcP care in vulnerable patients (older adults, organ failure, culturally and diverse populations, homelessness and substance misuse) is limited. The group recommended inclusive trial designs and low-burden methodologies to generate applicable data for these populations. Furthermore, given concerns about opioid misuse, current screening tools developed for chronic non-malignant pain may not be appropriate for use in advanced cancer. Interdisciplinary qualitative work and consensus methods are needed to define misuse in this context and to inform balanced risk management strategies, risk screening and interventions for those with opioid misuse issues.

Early engagement and formal collaboration between industry and palliative care professional bodies during decisions about opioid supply and production have been insufficient, reducing the ability of professional bodies to respond promptly. Inconsistent policies and regulations across states and local health districts also restrict access to specific ROOs formulations. The group recommended establishing mechanisms for rapid notification of anticipated drug shortages at international, national, state and local health service levels and developing ethical, timely and transparent liaison pathways among clinicians, researchers, pharmaceutical companies, regulators and pharmacies to ensure reliable, sustainable access. Finally, clear avenues for palliative care clinicians to advocate effectively for timely and equitable access to essential BTcP analgesia should be explored and implemented.

## Discussion

This gap analysis highlights persistent and actionable shortcomings in the recognition, assessment and management of BTcP in the Australian setting. Despite clinical need, high prevalence and substantial functional impact, practice remains fragmented by inconsistent definitions, limited BTcP assessment tools, differences in approaches, logistical barriers to ROOs implementation and inequitable access for vulnerable populations. The working group’s findings underscore that these problems are interdependent, and a more consistent, personalised and system-supported approach is needed.

A foundational challenge lies in the lack of a standardised definition for BTcP [9, 35]. As reported by the participants, existing guidelines vary in their inclusion of BTcP and/or the recognition of its subtypes. This heterogeneity hinders consistent clinical decision-making and limits the ability to compare outcomes among patients with BTcP in research. Consensus-based definitions developed through qualitative research and consensus methods such as Delphi and nominal group techniques involving key stakeholders are urgently needed to guide uniform practice and policy development. Meanwhile, there is a lack of BTcP-specific instrument, with the only instrument (BAT) having limited clinical utility due to concerns around questionnaire burden and lack of actionable guidance [34, 36]. Future studies should explore the development of assessment tools that incorporate the ability to distinguish between BTcP subtypes and support management aligned with the underlying pain mechanisms to optimise analgesic responses and reduce toxicity [37]. To achieve this, individuals with palliative care needs that are often excluded from clinical research due to their frailty, medical complexity and logistical challenges need to be included in research through innovative designs (e.g. aggregated n-of-1 and adaptive designs) and supportive strategies (e.g. pre-consent and memory aids) [38, 39].

While there is a need to study clinicians’ perspectives and approaches to BTcP assessment and management, this study supports clinician training to enhance awareness of BTcP and its potential differences from other cancer pain types [40]. Clinicians should avoid oversimplifying pain management and appropriately differentiate pain types and their interventions whilst supporting evidence is being generated [41]. At the health service level, successful implementation of BTcP interventions, such as the use of ROOs, will require co-design processes involving key stakeholders (e.g. multidisciplinary team members, pharmacists and executives) and alignment of the implementation plan with relevant regulatory guidance to ensure both safety and feasibility.

Access to opioids for cancer pain varies substantially across countries [42], with the contrast between Australia and the United States well documented. Australia operates within a highly regulated, centralised framework designed to balance safety with access. Opioid prescribing is governed by the Pharmaceutical Benefits Scheme, real-time prescription monitoring and state-based authority requirements [43]. In contrast, in the United States, opioid availability is less regulated, shaped by a fragmented healthcare system and variable insurance coverage. International comparisons consistently show that Australia’s regulatory environment results in more conservative opioid utilisation [44]. These differences underscore the need for context-specific strategies: Australia must ensure timely, equitable access within a tightly controlled system, while the United States continues to navigate the tension between preventing misuse and maintaining adequate analgesia for people with cancer.

Finally, recent and repeated national shortages of commonly used opioids underscore a lack of coordinated engagement between pharmaceutical industries and clinical stakeholders. There is a clear need for mechanisms for communication and contingency planning with opioid shortages at the hospital and community levels. This study supports the need for health services and policymakers to consider strategies to enhance collaboration among clinicians, professional bodies, researchers, industry and government regulatory bodies. Professional bodies and clinicians must be supported to advocate effectively for consistent, equitable access to these essential medications.

Altogether, these findings reinforce the need for coordinated clinical, research and national leadership to standardise the assessment of BTcP, facilitate safe and appropriate prescribing and ensure equitable opioid access to improve pain outcomes for patients with advanced cancer.

### Strengths and limitations of the study

This study has several strengths. The participants included medical and nursing clinicians and researchers with expertise in BTcP drawn from diverse geographic settings across Australia, including metropolitan and regional/rural locations. Data were collected through three roundtable sessions over 12 months, and content validity was repeatedly confirmed with participants by circulating and verifying meeting notes. These iterative discussions and member checks strengthened the credibility and trustworthiness of the findings.

There were also important limitations. The analysis reflects an Australian perspective and may have limited generalisability to countries with different regulatory frameworks, opioid funding mechanisms and availability of palliative care services. Although a targeted literature review informed the gap analysis, a formal systematic review was not undertaken, which could have introduced selection bias into the cited evidence. A gap analysis is a valuable strategic tool, but it has several inherent limitations. Its findings reflect the perspective of the individuals involved rather than a fully representative view. Whilst it identifies discrepancies and proposes conceptual solutions, it does not test their feasibility or effectiveness in practice.

Whilst the insights of the 13 members of the working group may provide a strong foundation for further work, they do not represent a formal national consensus and should be used to inform broader stakeholder engagement and Delphi processes. Finally, the study did not address the full complexity of the pain experience, as addressing psychological, spiritual, social and cultural modulators was outside its scope. Incorporating these perspectives, as well as patient and caregiver representation, will be essential to ensure that recommendations are person-centred, feasible and acceptable across diverse populations.

## Conclusion

It is essential to develop a consensus for the optimal approach to BTcP assessment and management. Within the context of local health service resources and policies, a personalised approach to research and care of individuals with various subtypes of BTcP is needed. Improved collaboration among clinicians, researchers, the pharmaceutical industry and government regulatory bodies may help address identified gaps, enhance required opioid access and advance research and evidence-based BTcP care in Australia.

## Data Availability

No datasets were generated or analysed during the current study.

## References

[1] Snijders RAH, Brom L, Theunissen M, van den Beuken- Everdingen MHJ (2023) Update on prevalence of pain in patients with cancer 2022: a systematic literature review and meta-analysis. cancers (Basel). 10.3390/cancers15030591 10.3390/cancers15030591 PMC9913127 36765547

[2] Greco MT, Roberto A, Corli O, Deandrea S, Bandieri E, Cavuto S, Apolone G (2014) Quality of cancer pain management: an update of a systematic review of undertreatment of patients with cancer. J Clin Oncol 32:4149–4154 25403222 10.1200/JCO.2014.56.0383

[3] van den Beuken- Everdingen MHJ, de Rijke JM, Kessels AG, Schouten HC, van Kleef M, Patijn J (2007) High prevalence of pain in patients with cancer in a large population-based study in The Netherlands. Pain. 10.1016/j.pain.2007.08.022 10.1016/j.pain.2007.08.022 17916403

[4] Gutgsell T, Walsh D, Zhukovsky DS, Gonzales F, Lagman R (2003) A prospective study of the pathophysiology and clinical characteristics of pain in a palliative medicine population. Am J Hosp Palliat Med 20:140–148 10.1177/104990910302000213 12693647

[5] Bennett MI, Kaasa S, Barke A, Korwisi B, Rief W, Treede R-D (2019) The IASP classification of chronic pain for ICD-11: chronic cancer-related pain. Pain 160:38–44 30586069 10.1097/j.pain.0000000000001363

[6] Mercadante S (2015) Breakthrough pain in cancer patients: prevalence, mechanisms and treatment options. Curr Opin Anaesthesiol 28:559–564 26263120 10.1097/ACO.0000000000000224

[7] Deandrea S, Corli O, Consonni D, Villani W, Greco MT, Apolone G (2014) Prevalence of breakthrough cancer pain: a systematic review and a pooled analysis of published literature. J Pain Symptom Manage 47:57–76 23796584 10.1016/j.jpainsymman.2013.02.015

[8] Li Q, Sheng Y, Liu X, Li J, Zhu L, Yang Y, Hu L (2025) A scoping review of breakthrough cancer pain: multidimensional patient needs and influencing factors. Asia Pac J Oncol Nurs 12:100780 41541009 10.1016/j.apjon.2025.100780 PMC12802105

[9] Yeo J (2024) Breakthrough pain and rapid-onset opioids in patients with cancer pain: a narrative review. J Yeungnam Med Sci 41:22–29 37424088 10.12701/jyms.2023.00367 PMC10834265

[10] Saini A, Tucci M, Tampellini M, Maina D, Bouraouia K, Giuliano P, Termine A, Castellano M, Campagna S, Laciura P (2013) Circadian variation of breakthrough pain in cancer patients. Eur J Pain 17:264–270 22715071 10.1002/j.1532-2149.2012.00184.x

[11] Mercadante S, Masedu F, Valenti M, Aielli F (2019) Breakthrough pain in patients with head & neck cancer. A secondary analysis of IOPS MS study. Oral Oncol 95:87–90 31345399 10.1016/j.oraloncology.2019.06.006

[12] Mercadante S, Adile C, Masedu F, Valenti M, Aielli F (2019) Breakthrough cancer pain in patients with abdominal visceral cancer pain. J Pain Symptom Manage 57:966–970 30822530 10.1016/j.jpainsymman.2019.02.014

[13] Crispo A, Luongo A, Nocerino D, Cascella M, Crisci M, Bifulco F, Schiavo D, Marchesini M, Coluccia S, Prete M, Amore A, Celentano E, Bimonte S, Cuomo A (2025) Assessment of breakthrough cancer pain among female patients with cancer: knowledge, management and characterization in the IOPS-MS study. Anticancer Res 45:3149–3164 40578946 10.21873/anticanres.17678

[14] Portenoy RK, Hagen NA (1990) Breakthrough pain: definition, prevalence and characteristics. Pain 41:273–281 1697056 10.1016/0304-3959(90)90004-W

[15] Davies AN, Dickman A, Reid C, Stevens A-M, Zeppetella G (2009) The management of cancer-related breakthrough pain: recommendations of a task group of the Science Committee of the Association for Palliative Medicine of Great Britain and Ireland. Eur J Pain 13:331–338 18707904 10.1016/j.ejpain.2008.06.014

[16] Jara C, Del Barco S, Grávalos C, Hoyos S, Hernández B, Muñoz M, Quintanar T, Meana JA, Rodriguez C, de Las Peñas R (2018) SEOM clinical guideline for treatment of cancer pain (2017). Clin Transl Oncol 20:97–107 29127593 10.1007/s12094-017-1791-2 PMC5785609

[17] Bossi P, Escobar Y, Pea F (2022) Rapid-onset opioids for management of breakthrough cancer pain: considerations for daily practice. Frontiers in pain research (Lausanne, Switzerland) 3:893530 35721659 10.3389/fpain.2022.893530 PMC9204512

[18] Brant J, Rodgers B, Gallagher E, Sundaramurthi T (2017) Breakthrough cancer pain: a systematic review of pharmacologic management. Clin J Oncol Nurs 21:71–80 28524907 10.1188/17.CJON.S3.71-80

[19] Mercadante S, Casuccio A, Noce G, Adile C (2025) Personalised breakthrough pain goals and responses in advanced cancer patients. Eur J Pain 29:e70103 40852775 10.1002/ejp.70103

[20] Davies AN, Elsner F, Filbet MJ, Porta-Sales J, Ripamonti C, Santini D, Webber K (2018) Breakthrough cancer pain (BTcP) management: a review of international and national guidelines. BMJ Support Palliat Care 8:241–249 29875184 10.1136/bmjspcare-2017-001467

[21] Cascella M, Monaco F, Nocerino D, Chinè E, Carpenedo R, Picerno P, Migliaccio L, Armignacco A, Franceschini G, Coluccia S, Gennaro PD, Tracey MC, Forte CA, Tafuri M, Crispo A, Cutugno F, Vittori A, Natoli S, Cuomo A (2022) Bibliometric network analysis on rapid-onset opioids for breakthrough cancer pain treatment. J Pain Symptom Manage 63:1041–1050 35151801 10.1016/j.jpainsymman.2022.01.023

[22] Fallon M, Giusti R, Aielli F, Hoskin P, Rolke R, Sharma M, Ripamonti C (2018) Management of cancer pain in adult patients: ESMO clinical practice guidelines. Ann Oncol 29:iv166–iv191 30052758 10.1093/annonc/mdy152

[23] Swarm RA, Abernethy AP, Anghelescu DL, Benedetti C, Buga S, Cleeland C, Deleon-Casasola OA, Eilers JG, Ferrell B, Green M, Janjan NA, Kamdar MM, Levy MH, Lynch M, McDowell RM, Moryl N, Nesbit SA, Paice JA, Rabow MW, Syrjala KL, Urba SG, Weinstein SM, Dwyer M, Kumar R (2013) Adult cancer pain: clinical practice guidelines in oncology. J Natl Compr Canc Netw 11:992–1022 23946177 10.6004/jnccn.2013.0119 PMC5915297

[24] Paice JA, Bohlke K, Barton D, Craig DS, El-Jawahri A, Hershman DL, Kong LR, Kurita GP, LeBlanc TW, Mercadante S, Novick KLM, Sedhom R, Seigel C, Stimmel J, Bruera E (2023) Use of opioids for adults with pain from cancer or cancer treatment: ASCO guideline. J Clin Oncol 41:914–930 36469839 10.1200/JCO.22.02198

[25] Cancer Council Australia (2024) Cancer pain management in adults. In: Editor (ed)^(eds) Book Cancer pain management in adults, City

[26] López Alarcón MD, Villegas Estévez F, Contreras Martínez J, Ferrer Albiach C, Pastor Peidro JR, Terol Casterá MJ, Velázquez Rivera I (2025) Consensus on the diagnosis and management of patients with breakthrough cancer pain (BTcP), including procedural BTcP: diopro study. J Pain Palliat Care Pharmacother 39:477–489 40823980 10.1080/15360288.2025.2546104

[27] Crawford GB, Lakhani A, Palmer L, Sebalj M, Rolan P (2023) A systematic review of qualitative research exploring patient and health professional perspectives of breakthrough cancer pain. Support Care Cancer 31:619 37812248 10.1007/s00520-023-08076-9 PMC10562491

[28] Duzova US, Kilic M, Gundogdu F, Yildirim D, Can G, Talu GK (2025) Breakthrough cancer pain: assessment and self-management perspectives among oncology nurses. BMC Nurs 24:1178–1111 40999422 10.1186/s12912-025-03827-x PMC12465389

[29] Crawford GB, Lakhani A, Palmer L, Sebalj M, Rolan P (2024) Breakthrough cancer pain management: mixed-methods study of health care professionals. BMJ Support Palliat Care. 10.1136/spcare-2024-004951 39658093 10.1136/spcare-2024-004951

[30] Lauder B (2013) Conducting a gap analysis. In: Levin RF, Feldman HR (eds) Teaching evidence-based practice in nursing. Springer Publishing Company, New York, pp 75–84

[31] Daveson BA, Allingham SF, Clapham S, Johnson CE, Currow DC, Yates P, Eagar K (2021) The PCOC symptom assessment scale (SAS): a valid measure for daily use at point of care and in palliative care programs. PLoS One 16:e0247250 10.1371/journal.pone.0247250 PMC7993777 33765077

[32] Hearn J, Higginson IJ (1999) Development and validation of a core outcome measure for palliative care: the palliative care outcome scale. BMJ Quality & Safety 8:219–227 10.1136/qshc.8.4.219 PMC2483665 10847883

[33] Poquet N, Lin C (2016) The brief pain inventory (BPI). J Physiother 62:52 26303366 10.1016/j.jphys.2015.07.001

[34] Webber K, Davies AN, Zeppetella G, Cowie MR (2014) Development and validation of the breakthrough pain assessment tool (BAT) in cancer patients. J Pain Symptom Manage 48:619–631 24766740 10.1016/j.jpainsymman.2013.10.026

[35] Greenfield K, Schoth DE, Hain R, Bailey S, Mott C, Rajapakse D, Harrop E, Renton K, Anderson A-K, Carter B (2024) A rapid systematic review of breakthrough pain definitions and descriptions. Br J Pain 18:215–226 10.1177/20494637231208093 PMC11092936 38751563

[36] Hagen NA, Stiles C, Nekolaichuk C, Biondo P, Carlson LE, Fisher K, Fainsinger R (2008) The Alberta breakthrough pain assessment tool for cancer patients: a validation study using a Delphi process and patient think-aloud interviews. J Pain Symptom Manage 35:136–152 18178370 10.1016/j.jpainsymman.2007.03.016

[37] Davis MP (2011) Breakthrough pain in cancer patients— characteristics, impact, and assessment. Oncology & Hematology Review (US). 10.17925/ohr.2011.07.1.12

[38] Nikles J, Mitchell GK, Schluter P, Good P, Hardy J, Rowett D, Shelby-James T, Vohra S, Currow D (2011) Aggregating single patient (n-of-1) trials in populations where recruitment and retention was difficult: the case of palliative care. J Clin Epidemiol 64:471–480 20933365 10.1016/j.jclinepi.2010.05.009

[39] Pallmann P, Bedding AW, Choodari-Oskooei B, Dimairo M, Flight L, Hampson LV, Holmes J, Mander AP, Sydes MR, Villar SS (2018) Adaptive designs in clinical trials: why use them, and how to run and report them. BMC Med 16:1–15 10.1186/s12916-018-1017-7 PMC5830330 29490655

[40] Batistaki C, Graczyk M, Janecki M, Lewandowska AA, Moutinho R, Vagdatli K (2022) Relationship between breakthrough cancer pain, background cancer pain and analgesic treatment–case series and review of the literature. Drugs Context. 10.7573/dic.2022-9-4 36660261 10.7573/dic.2022-9-4 PMC9828877

[41] Mercadante S, Cuomo A (2016) Breakthrough cancer pain: ten commandments. Value Health 19:531–536 27565269 10.1016/j.jval.2016.03.002

[42] International Narcotics Control Board (2016) Availability of internationally controlled drugs: ensuring adequate access for medical and scientific purposes: indispensable, adequately available and not unduly restricted. UN, Vienna

[43] Department of Health, Disability, and Ageing (Australian Government), (2026) Pharmaceutical benefits scheme (PBS). Pharmaceutical Benefits Scheme (PBS), City

[44] Berterame S, Erthal J, Thomas J, Fellner S, Vosse B, Clare P, Hao W, Johnson DT, Mohar A, Pavadia J (2016) Use of and barriers to access to opioid analgesics: a worldwide, regional, and national study. Lancet 387:1644–1656 26852264 10.1016/S0140-6736(16)00161-6

[45] National Health and Medical Research Council (2023) National statement on ethical conduct in human research. National statement on ethical conduct in human research. National Health and Medical Research Council, City

